# The quality of medicines for the prevention and management of hypertensive disorders of pregnancy: A systematic review

**DOI:** 10.1371/journal.pgph.0002962

**Published:** 2024-02-27

**Authors:** Pooja Maharjan, Meghna Prasannan Ponganam, Pete Lambert, Joshua P. Vogel, Michelle McIntosh, Annie McDougall

**Affiliations:** 1 Monash Institute of Pharmaceutical Sciences, Monash University, Parkville, Australia; 2 Burnet Institute, Maternal, Child and Adolescent Health Program, Melbourne, Australia; 3 School of Public Health and Preventive Medicine, Monash University, Melbourne, Australia; PLOS: Public Library of Science, UNITED STATES

## Abstract

The quality of medicines for the prevention and management of hypertensive disorders of pregnancy globally is a critical challenge in the reduction of maternal mortality rate. We aimed to conduct a systematic review of available studies on the quality of the eight medicines recommended globally for the prevention and management of hypertensive disorders of pregnancy. We searched five electronic databases- Ovid MEDLINE, EMBASE, CINAHL, ProQuest and Cochrane Library, and also grey literature, without year or language limitations. Any study assessing the quality parameters (Active Pharmaceutical Ingredients, pH, sterility, solubility, impurities) of medicines by using any valid laboratory methods was eligible. Two reviewers independently screened the studies, extracted data and applied Medicine Quality Assessment Reporting Guidelines tool for quality assessment. Results were narratively reported and stratified by the drug types. Of 5669 citations screened, 33 studies from 27 countries were included. Five studies reported on the quality of magnesium sulphate—two (Nigeria and USA) found substandard medicine due to failing API specification and contaminants, respectively. Another study from Nigeria and a multi-country study (10 lower-middle- and low-income countries) found poor-quality due to failing the pH criteria. Seven of eight studies evaluating aspirin found quality issues, including degraded medicines in five studies (Brazil, USA, Yugoslavia and Pakistan). Five studies of calcium supplements found quality issues, particularly heavy metal contamination. Of 15 antihypertensives quality studies, 12 found substandard medicines and one study identified counterfeit medicines. This systematic review identified pervasive issues of poor-quality medicines across all recommended medicines used to prevent or treat hypertensive disorders of pregnancy, raising concerns regarding their safety and effectiveness.

## Introduction

Globally, an estimated 287,000 women die each year due to complications during or following pregnancy and childbirth, resulting in a global maternal mortality ratio (MMR) of 223 maternal deaths per 100,000 live births [1]. Of these, 95% occurred in low-and middle-income countries (LMICs), with the highest MMRs in Sub-Saharan African and South Asian countries [1]. Hypertensive disorders of pregnancy (HDP), the second leading direct cause of maternal mortality, are estimated to account for 27,830 maternal deaths each year [2]. They are a heterogenous group of disorders, including chronic hypertension, gestational hypertension, preeclampsia/eclampsia and preeclampsia superimposed on chronic hypertension [3].

Management of HDP involves the use of medicines repurposed from other hypertensive conditions, which remain off-label for use in pregnant women [4]. The World Health Organization (WHO) and National Institute for Health and Care Excellence (NICE) guidelines recommend that women at increased risk of preeclampsia receive low-dose aspirin from 12 weeks of gestation, onwards [5,6]. They also recommend calcium supplementation (1.5–2 gram/day orally) to women living in regions with low dietary calcium intake [6,7]. Antihypertensive medicines, such as labetalol, nifedipine, methyldopa, hydralazine, amlodipine and enalapril, are recommended for hypertension management depending on the severity [5,6,8]. Magnesium sulphate is recommended for the prevention and management of eclamptic seizures [5,6].

Access to good-quality medicines is critical for the management of HDP [9]. According to WHO, 10.5% of all available medicines for any condition are substandard or falsified globally, and this is more prevalent in low- and middle-income countries (LMICs) [10]. Substandard medicines are manufactured by registered pharmaceutical companies, but fail to meet quality specifications [11,12]. Falsified medicines are those that are manufactured and packaged to purposively deceive consumers, and may contain poor quality ingredients or no active ingredients at all [11,12]. Previous systematic reviews have identified evidence of poor-quality in medicines used for pregnancy complications, including postpartum haemorrhage and preterm birth [13,14]. Ineffective medicines thus present a major challenge to reaching the Sustainable Development Goals of ending preventable maternal and newborn mortality [12].

Several studies have been published on the quality of medicines used for HDP, however their findings have not been evaluated systematically. One previous systematic review reported on some maternal medicines including magnesium sulphate [15] without considering other medicines for HDP. Therefore, this review aimed to identify, critically appraise and synthesize the findings of studies on the quality of commercially available medicines recommended for prevention and treatment of HDP.

## Methods

This review followed the standards of the Preferred Reporting Items for Systematic Reviews and Meta-Analyses (PRISMA) guidelines and the Cochrane Handbook for Systematic Reviews. The protocol was preregistered on PROSPERO (CRD42023425055).

We pre-specified nine medicines (**Table 1**) on the basis of current WHO and NICE guidelines on the prevention and management of HDP. Detail of inclusion and exclusion criteria are mentioned in the appendix (see **S2 Text**). Outcome of interest for this review included multiple parameters used to assess medicine quality, including any one or more of macroscopic appearance, extractable volume, pH range, active pharmaceutical ingredient (API) content, sterility, solubility, impurities or related substances, as well as packaging, labelling, storage and transportation conditions. We reported failure rates on the quality of studied medicines according to the parameters assessed by the authors against the pharmacopoeia specification (whether it was International Pharmacopoeia, US Pharmacopoeia, British Pharmacopoeia or other relevant Pharmacopoeia) or the manufacturer standard of each included study. Medicines were considered to be substandard if they did not comply with these standards on specified API content, impurities, or containing related substances. Medicines were considered to be falsified (or counterfeit) if they had no active ingredients, and were considered falsified by the authors of the study in question.

**Table 1:** Recommended medicines for the prevention and treatment of hypertensive disorders of pregnancy.

DRUG	DRUG CATEGORY	CLINICAL USE	DOSAGE	GUIDELINE
Calcium	Dietary supplements	Prevention	1.5–2 g/day; oral	WHO [7] & NICE [6]
Aspirin	Antiplatelet agents	Prevention	75mg/day; oral 75-150mg/day; oral	WHO [5] & NICE [6]
Labetalol	β-blockers	Management of hypertension	200–1200 mg/day; oral 20–40 mg; IV	WHO [8] & NICE [6]
Nifedipine	Calcium channel blockers	Management of hypertension	10–30 mg/day; oral	WHO [8] & NICE [6]
Methyldopa	Centrally acting α2-adrenergic agonists	Management of hypertension	0.5 g/day; oral	WHO [5] & NICE [6]
Hydralazine	Direct vasodilators	Management of hypertension	50–300 mg/day; IV	WHO [5] & NICE [6]
Amlodipine	Calcium channel blockers	Management of postpartum hypertension	10 mg/day; oral	NICE [6]
Enalapril	ACE inhibitors	Management of postpartum hypertension	10 mg/day; oral	NICE [6]
Magnesium Sulphate	Anticonvulsant	Prevention and management of eclamptic seizures	Loading dose: 4g IV followed by 10g IM immediately, then 5g IM in every 4 hours	WHO [5] & NICE [6]

### Electronic databases and searching methods

A comprehensive search of five electronic databases–Ovid MEDLINE, EMBASE, CINAHL, ProQuest and Cochrane Library–was conducted on 28^th^ October 2022. The search strategy was created with the help of an experienced librarian (see **S1 Text**). The search was complemented by reviewing the reference lists of all included articles. Grey literature searches included unpublished studies identified through contacts with investigators who had conducted the studies. All recovered citations were uploaded into EndNote, and then screened using Covidence. Title and abstract screening, and subsequent full-text review was conducted by two independent reviewers, with conflicts resolved via discussion or consulting a third reviewer. Reports in languages other than English were first screened with an online translation tool (Google Translate) and subsequently translated by a native speaker to confirm eligibility and for data extraction and quality assessment.

### Data extraction and quality assessment

Data for this review were extracted from included studies using Covidence. Variables of interest were country, sample collection year, types of outlets where samples were collected, country of manufacture, types of tests performed, total number of samples assayed, which pharmacopoeia or standard method was used and data relevant to outcomes of interest.

We assessed the quality of all included studies using the Medicine Quality Assessment Reporting Guidelines (MEDQUARG) checklist for reports of surveys of the quality of medicine [16]. Each study was graded on following domains: (1) survey details, (2) definition of substandard, falsified and degraded medicines, (3) types of outlets sampled, (4) sampling design and sample calculation, (5) type and dosage of units collected per outlet, (6) random sampling, (7) samplers’ detail, (8) packaging assessment, (9) statistical methods, (10) chemical analysis description, (11) method validation details and (12) blinding or chemical assessors. There is no established threshold using the MEDQUARG tool to define study quality, hence the studies were categorised using the approach of Torloni et al., which similarly evaluated studies on the quality of maternal medicines [13], where good methodological quality was defined as those with MEDQUARG score ≥ 6, and a score < 6 as low methodological quality. Two reviewers performed data extraction and quality assessment independently, with conflicts resolved via discussion or consultation with a third reviewer.

### Data synthesis and statistical analyses

Data were presented descriptively. We reported on the prevalence of failed samples due to API content (whether too low or too high), sterility, pH and presence of impurities or related substances. Included studies and extracted data were organised by medicine type. We also reported the country where the study was conducted, and categorised that country income level, using the World Bank 2022 classification, and by type of outlets where the samples were collected.

## Results

**Fig 1:**
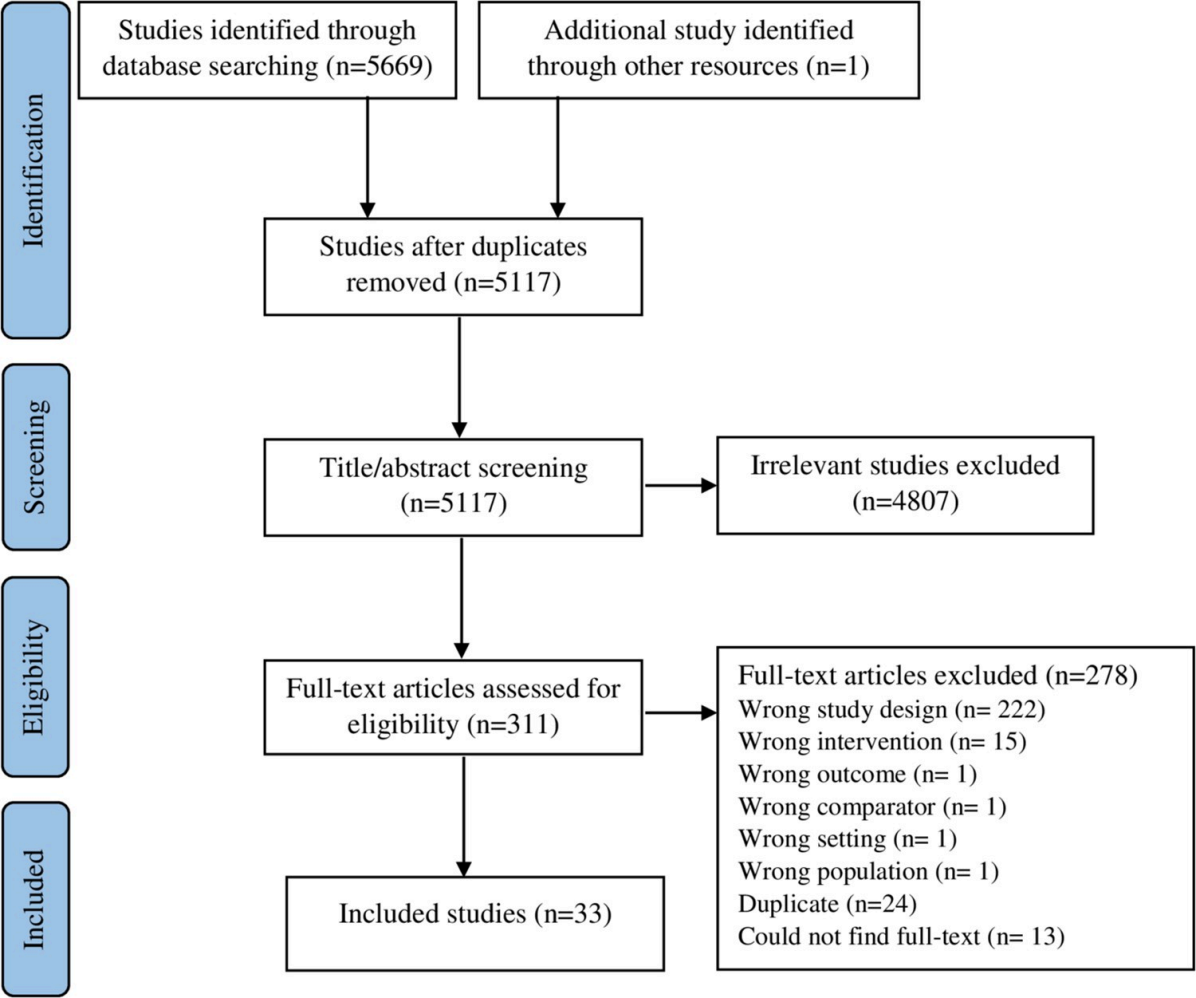
PRISMA Flow diagram illustrating the process of study identification and selection.

A total of 5669 citations were identified for screening. Of these, 278 studies were excluded during full-text screening. In total, 33 studies were included in this review (**Fig 1**). This included one article published as a research letter [17], and an unpublished report from Ethiopia [18]; the remaining articles were published in peer-reviewed journals.

The 33 included studies assessed the quality of different medicines–magnesium sulphate (five studies) (**Table 2**) [17–21], aspirin (eight studies) (**Table 3**) [22–29], calcium supplements (five studies) (see **Table 4**) [30–34], and antihypertensive medicines–amlodipine (seven studies), nifedipine (two studies), methyldopa (three studies), enalapril (three studies) and hydralazine (one study) (see **Table 5**) [35–49]. No study assessing quality of labetalol was identified. Studies were conducted between 1979–2021 and in a wide range of countries: three studies in low-income countries (LICs), 14 in upper-middle income countries (UMICs) and eight in high-income countries (HICs). Nineteen studies reported the setting from where medicines were sampled. This included patent medicine stores, wholesale and retail pharmacy stores, midwifery clinics, primary health care centres, state hospital, pharmacy depot, warehouses, compounding pharmacies, central medical stores, NGO central stores, basic pharmacies, pharmaceutical companies, registered and unregistered pharmacies, informal drug stores, and pharmacy chain stores. In total, 23 (69.7%) studies were of good methodological quality (score ≥ 6).

**Table 2:** Characteristics and findings of studies on quality of magnesium sulphate.

Study	Country	Income level of country	Year of sample collection	Pharmacopoeia / standard methods	Study quality grade	Total N of samples assayed	Region / Country of manufacture (N of samples)	N of samples from different level settings	Test performed	Microbial contaminant found (Y/N)	Percent failed samples with physical parameters	API content failed samples n (%)	Percent failed samples with pH	Poor-quality (YES/NO) Comments
Anyakora, 2018 [19]	Nigeria	LMIC	May 23–28, 2016	USP 39 and BP 2016	>10	163	No information	5 patent medicine stores, 4 wholesale and retail pharmacy stores, 5 Midwife clinics, 5 primary health care centres, 1 state hospital stores, 1 FMC warehouse	Titrimetric method (API assay), pH, sterility test	N	Physical parameters were not measured	4 (2.4%)	0 (0.0%)	YES Due to not meeting the monograph specific range for API content (93% to 107%)
Boyce, 2014 [17]	USA	HIC	Mid-March through mid-June 2013	No information	6	1070	USA	Compounding pharmacy	DNA sequencing (sterility)	Y	Discovered floaters in a bag of Magnesium sulphate	Not measured	Not measured	YES Due to fungal contamination of *Hamigera insecticola*, *Neosartrua hiratsukae*, *Penicillium chrysogenum*, *Penicillin rubens*
UNCoLSC, 2015[50]	Burkina Faso, Kenya, Madagascar, Nepal, Nigeria, Tajikistan, Tanzania, Uganda, Vietnam and Zimbabwe	LIC and LMIC	September—November 2013	International Pharmacopoeia 4th Edition	>10	19	India, Vietnam	central medical stores, NGO central stores, warehouse of importers or major distributors, or other facilities	Appearance, pH analysis, content assay	Sterility-not tested	0 (0.0%)	0 (0.0%)	2 (10.5%)	YES Due to not complying with pH specification
Zelalem, 2015 [18]	Ethiopia	LIC	April—July 2015	USP	> 10	13	Korea and Germany	Regulated public medicine outlets, regulated private medicine outlets	API content assay, pH analysis	Sterility-not tested	Physical parameters were not measured	0 (0.0%)	0 (0.0%)	NO However, all samples were not registered
Angus, 2021 [21]	Nigeria	LMIC	May–July 2019	USP	>10	90	No information	Drug outlets	Visual analysis, API content assay, pH analysis, sterility test	N	0 (0.0%)	0 (0.0%)	40 (44.4%)	YES Due to not complying with pH specification

HIC: High income countries, LMIC: Low and middle-income countries, LIC: Low income countries.

**Table 3:** Characteristics and findings on studies of quality of aspirin.

Study	Country	Income level of country	Year of sample collection	Pharmacopoeia / standard methods	Study quality grade	Total N of samples assayed	Region/ Country of manufacture (N of samples)	N of samples from different level settings	Test performed	Percent failed samples with physical parameters	API content failed samples n (%)	Failed samples with impurities or related substances	Poor-quality (YES/NO) Comments
Audu, 2012 [22]	Nigeria	LMIC	No information	USP 2007	>10	10	Nigeria	Pharmacy shops	HPLC-UV spectrophotometer (API assay)	Not measured	2 (20.0%)	Not measured	YES Due to not meeting the monograph specific range for API content (90% to 110%)
Bunhak, 2010 [23]	Brazil	UMIC	June 2004—March 2006	USP 23rd edition (identification and dissolution) BP 1999 (API and impurities assay) Brazilian pharmacopoeia 4th edition (physical tests-uniformity of mass, hardness, friability and disintegration time)	6	8	Brazil	Basic pharmacy	Uniformity of mass, hardness, friability, disintegration time, neutralization titration, free salicylic acid limit test, dissolution test	6 (75%)	0 (0.0%)	5 (62.5%)	YES Due to not meeting physicochemical parameters and also detection of free salicylic acid above the acceptance range
Melo, 2006 [24]	Brazil	UMIC	2003	USP 23rd Edition (content assay), BP (free salicylic acid assay), Brazilian Pharmacopoeia (physical parameters)	9	1000	No information	Basic pharmacy	Physical screening test, colorimetric and volumetric assay (content and impurity assay)	Not reported	Not mentioned	Not reported	YES Due to not complying with the standard specification in terms of colour, appearance, odour and free salicylic acid content
Juhl, 1979 [25]	USA	HIC	No information	USP XIX	3	170	USA	Pharmaceutical company	HPLC (content assay)	Not measured	44 (25.9%)	44 (25.9%)	YES Due to not meeting the monograph specific range for API content and detection of free salicylic acid above the acceptance range
Kirchhoefer, 1979 [26]	USA	HIC	No information	No information	3	172	USA	Pharmaceutical companies	HPLC and spectrophotometry (impurities assay)	Not measured	Not measured	22(12.8%)	YES Due to detection of free salicylic acid above the acceptance range
Nikolić, 1994 [27]	Yugoslavia	UMIC	No information	USP 21	2	34	No information	No information	HPLC (API content assay and impurities content assay)	Not measured	11 (32.4%)	19 (55.9%)	YES Due to not meeting monograph specific range for API content, detection of salicylic acid above the acceptance range and 11 of poor-quality aspirin were expired
Saeed, 2018 [28]	Iraq	UMIC	No information	No information	7	20	Iraq, UK, Turkey, Syria, Netherlands, Lebanon, Romania, Egypt, Atlanta, USA	No information	HPLC (content assay), atomic absorption spectrometry (heavy metal ion assay)	Not measured	0 (0.0%)	0 (0.0%)	NO
Yang, 2004 [29]	Cambodia	LMIC	October 2022—February 2023	USP 20 and USP 25	>10	96	Thailand, Vietnam, India, Malaysia, USA	76 legal and 20 illegal drug stores from seven districts of Phnom Penh	Physical parameters, content assay, dissolution test	2 (2.1%) failed in hardness test, 1(1.0%) failed in weight variation, 16 (16.7%) failed in disintegration test, 32(33.3%) failed in friability test	16 (16.7%) failed in API content assay, 89 (92.7%) failed in dissolution test	Not measured	YES Due to not meeting monograph specific range for API content, and physicochemical parameters

HIC: High income countries, UMIC: Upper middle-income countries, LMIC: Low and middle-income countries.

**Table 4:** Characteristics and findings on studies of quality of calcium supplements.

Study	Country	Income level of country	Year of sample collection	Pharmacopoeia/ standard methods	Study quality grade	Total N of samples assayed	Region / Country of manufacture (N of samples)	N of samples from different level settings	Test performed	Microbial contaminants found (Y/N)	Failed samples with physical parameter (%)	API content failed samples n (%)	Failed samples with impurities or related substances	Poor-quality (YES/NO) Comments
Boulos, 1988 [30]	USA	HIC	No information	No information	4	10	No information	No information	Atomic absorption spectrometry (metal assay)	Sterility-not tested	Not measured	Not measured	1 (10.0%)	YES Due to detection of nine trace elements above the acceptance range
Levine, 2005 [31]	USA	HIC	No information	United States Environmental Protection Agency (UNESPA)	5	3	No information	No information	Atomic absorption spectrometry (mercury assay)	Sterility-not tested	Not measured	Not measured	1 (33.3%)	YES Due to detection of mercury above the acceptance range
Mattos, 2005 [32]	Brazil	UMIC	June 2003 –January 2004	Brazilian Pharmacopoeia 2000	10	23	Brazil and USA	Local and national chains of pharmacies	Atomic absorption spectrometry (calcium content assay) graphite furnace (lead content assay)	Sterility-not tested	Not measured	7 (31.1%)	14 (60.9%)	YES Due to not meeting monograph specific range for API content and detection of lead contamination above the acceptance range
Rehman, 2010 [33]	Pakistan	LMIC	No information	US Pharmacopoeia	6	27	USA, Switzerland, Pakistan, UK	Local markets in Islamabad	Atomic absorption spectrometry (lead content assay)	Sterility-not tested	Not measured	Not measured	24 (88.9%)	YES Due to detection of lead contamination above the acceptance range
Ross, 2000 [34]	USA	HIC	March, 2000	No information	7	22	USA	National chains of pharmacies	Electrothermal atomic absorption assay (lead content assay)	Sterility-not tested	Not measured	Not measured	8 (36.4%)	YES Due to detection of lead contamination above the acceptance range

HIC: High income countries, UMIC: Upper middle-income countries, LMIC: Low and middle-income countries.

**Table 5:** Characteristics and findings on studies of quality of antihypertensive medicines.

Study	Drug	Country	Income level of country	Year of sample collection	Pharmacopoeia / standard methods	Study quality grade	Total N of samples assayed	Region / Country of manufacture (N of samples)	N of samples from different level settings	Test performed	Percent failed samples with physical parameter n (%)	API content failed samples n (%)	Percent failed samples with impurities or related substances n (%)	Poor-quality (YES/NO) Comments
Antignac, 2017 [35]	Amlodipine	Benin, Burkina Faso, Congo (Brazzaville), the Democratic Republic of Congo, Guinea, Cote d’lvoire, Mauritania, Niger, Senegal, Togo	LIC and LMIC	November 2012-August 2014	International Conference on Harmonization	>10	305	Africa, Asia, Europe, Unknown	880 Licensed pharmacy, 650 unlicensed pharmacy (street market)	Validated reversed phase liquid chromatography with tandem mass spectrometry (API assay)	Not measured	87 (28.5%)	Not measured	YES Due to not meeting monograph specific range for API content
Dandamudi, 2019 [36]	Amlodipine besylate	India	LMIC	No information	USP 233	4	3	No information	No information	Inductively coupled plasma-mass spectrometry (heavy metal assay)	Not measured	Not measured	0 (0.0%)	NO Detected heavy element (As, Hg, Pb, Cd, Cr, Ni) were within the acceptance range
Eichie, 2011 [37]	Amlodipine besylate	Nigeria	LMIC	No information	BP 2003	10	10	Nigeria, UK, India, Portugal, Germany, Turkey	No information	API assay, Disintegration and dissolution test	0 (0.0%)	3 (30.0%)	Not measured	YES Due to not meeting monograph specific range for API content and were unregistered
Gyanwali, 2015 [38]	Amlodipine	Nepal	LMIC	No information	No information	9	5	Nepal, India	No information	Self-life, physical examination, average weight, HPLC for API assay	0 (0.0%)	0 (0.0%)	Not measured	NO
Olayemi, 2012 [39]	Amlodipine	Nigeria	LMIC	No information	USP and BP	10	8	No information	Registered pharmacy stores in the Lagos city	Physical parameters (friability and hardness test) test, dissolution test, UV spectrophotometry and HPLC	1 (12.50%) failed in friability test 2(25%) failed in hardness test	1 (12.5%)	Not measured	YES Due to not meeting monograph specific range for API content and physico-chemical parameters
Rahman, 2019 [40]	Amlodipine	Cambodia	LMIC	June 2011, June 2012 and August 2013	USP 34 and 35, BP 2012	>10	79	Not mentioned	Depot, Pharmacy, Wholesaler	Visual screening test for packaging and expiration dates and registration, HPLC (API content assay) and dissolution test	7 (8.9%) did not have registration label on the box	1 (1.3%) failed in API content 6(7.6%) failed in content uniformity and 2(2.5%) failed in dissolution test	Not measured	YES Due to not meeting monograph specific range for API content and physico-chemical parameters
Redfern, 2019 [41]	Amlodipine	Nigeria	LMIC	No information	USP	>10	195	India, United Kingdom, Nigeria, China, Portugal, Pakistan, Slovenia, Malaysia, Bangladesh, Germany and Egypt	22 pharmacy outlets from 6 local government areas	High performance liquid chromatography with photodiode array detection (API content assay)	Not measured	48 (24.6%)	Not measured	YES Due to not meeting monograph specific range for API content
Ndichu, 2018 [42]	Nifedipine	Nigeria	LMIC	May—July 2017	USP and International pharmacopoeia	>10	102	Asia, Europe, Africa, Unknown (India, Israel, Nigeria, Switzerland, Germany, Slovenia)	Registered pharmacies (private and public) from 6 local government level	Visual screening test, reverse-phase HPLC with mass spectrometry for API content assay and impurities content assay	0 (0.0%)	31(30.4%)	76 (74.5%)	YES Due to not meeting monograph specific range for API content and detection of related substance above the acceptance range
Feltkam, 1989 [43]	Nifedipine	Germany	HIC	June–October 1989	German Federal Health Authority and USP	4	68	Germany	Not specified	Light stability test, HPLC (content assay after in vitro release test), and dissolution test	27.9% packaged and 76.5% unpackaged tablets	60.%	Not measured	YES Due to not meeting monograph specific range for API content and light stability test
Twagirumukiza, 2009 [44]	Methyldopa	Rwanda	LIC	No information	USP 2006 and National Formulary	>10	3	Belgium, Kenya, Pakistan	4 public pharmacies (located in Kigali or Butare) and 34 randomly selected private pharmacies in Kigali	Visual screening test (colour and odour) and HPLC (API content assay)	Not measured	0 (0.0%)	Not measured	YES
Haruna, 2013 [45]	Alpha methyldopa	Nigeria	LMIC	No information	USP	7	4	No information	No information	Non-aqueous titration (API assay)	Not measured	1 (25.0%)	Not measured	YES Due to not meeting monograph specific range for API content
Lima, 2011 [46]	methyldopa	Brazil	UMIC	No information	Brazilian Pharmacopoeia 4^th^ Ed	4	20	No information	No information	Visual screening test spectrophotometry analysis (content assay)	0 (0.0%)	10 (50.0%)	Not measured	YES Due to not meeting monograph specific range for API content
	Enalapril maleate	Brazil	UMIC	No information	Brazilian Pharmacopoeia 4th Ed		20	No information	No information	Visual screening test spectrophotometry analysis (content assay and content uniformity assay)	0 (0.0%)	20 (100.0%)	Not measured	
Leal, 2014 [47]	Enalapril maleate	Brazil	UMIC	No information	USP 21st edition	7	5	No information	Local markets	Neutron active analysis (metal content assay)	Not measured	Not measured	5 (100.0%)	YES Due to detection of elements Al, Au, Br, Ce, Co Hf, La, Sb, Sc, Sm, Th, Zn, Cr above the acceptance range
Baloglu, 2001 [48]	Enalapril maleate	Turkey	UMIC	No information	USP XXII	3	8	No information	Pharmaceutical company	Physical parameters (hardness and friability) spectrophotometric method (content assay and dissolution test)	8 (100.0%)	0 (0.0%)	Not measured	YES Due to not meeting the specification for physico-chemical parameters
Matsui, 1982 [49]	Hydralazine	USA	HIC	No information	No information	3	16	No information	No information	GLC (hydrazine content assay)	Not measured	Not measured	12 (75.0%)	YES Due to detection of related substance, hydrazine above the acceptance range

HIC: High income countries, UMIC: Upper middle-income countries, LMIC: Low and middle-income countries, LIC: Low income countries.

Al: Aluminium, Au: Gold, Br: Bromine, Ce: Cerium, Co: Cobalt, Hf: Hafnium, La: Lanthanum, Sb: Antimony, Sc: Scandium, Sm: Samarium, Th: Thorium, Zn: Zinc, Cr: Chromium.

### Magnesium sulphate

Five studies assessed the quality of magnesium sulphate (**Table 2**), one from the USA (2014), two from Nigeria (2018 and 2021), one Ethiopian post-marketing surveillance report (2015) and one from the 2015 United Nations Commissions on Life-Saving Commodities (UNCoLSC) report that included 10 different countries- Burkina Faso, Kenya, Madagascar, Nepal, Nigeria, Tajikistan, Tanzania, Uganda, Vietnam and Zimbabwe [17–21]. The USA study found fungal contamination of *Hamigera insecticola*, *Neosartrua hiratsukae*, *Penicillium chrysogenum*, *Penicillin rubens* in a single bag of magnesium sulphate from a compounding pharmacy [17]. Two studies from Nigeria- 163 samples in the 2018 study and 90 samples in the 2021 study- reported that all samples passed sterility testing [19,21]. In the 2018 study, four of 163 samples did not meet API content specification and were reported as substandard [19]. In contrast, the 2021 Nigerian study [21] and post-marketing surveillance study from Ethiopia (13 samples) [18] reported all samples passed API content assays. The UNCoLSC study assessed a total 19 samples from 10 different countries in which two samples (one from Burkina Faso and one from Vietnam) failed to meet pH specifications [50]. Similarly, the Nigerian study (2021) reported 40 of 90 samples as poor-quality due to not meeting pH criteria [21]. Whereas the Nigerian (2018) and Ethiopian (2015) studies reported all samples passed the criteria for pH testing [18,19].

### Aspirin

A total of eight aspirin quality studies were included (**Table 3**) [22–29]. All but one study (Iraq, 20 samples [28]) found evidence of substandard aspirin. Four studies, two studies- both from the USA conducted in 1979 [25,26], one from Brazil (2010) [23] and one from Yugoslavia (1994) [27], detected free salicylic acid above the acceptance range in 22 samples (12.8%), 44 samples (25.9%), five samples (62.5%) and 19 samples (55.9%), respectively. Another Brazilian study (2006) also detected free salicylic acid in aspirin samples, however the number of failed samples were not disclosed [24]. Only three studies from Nigeria (2012) [22], Cambodia (2004) [29] and the USA (1979) [25] assessed API content of aspirin. These found that API specifications were not met in two samples (20.0%), 16 samples (16.7%) and 44 samples, respectively. A Brazilian study (2010) found six samples (75%) failed to meet the dissolution criteria [23]. In the Cambodian study, physicochemical parameters such as hardness, weight variation, disintegration, friability and dissolution were assessed under quality evaluation, in which two samples (2.1%), one sample (1.0%), 16 samples (16.7%) and 32 samples (33.3%) failed in hardness, weight variation, disintegration and friability test, respectively [29].

### Calcium supplements

Five studies assessed the quality of calcium supplements (see **Table 4**). All five studies tested for the presence of heavy metal impurities, such as lead or mercury [30–34]. Three studies from the USA (22 samples), Brazil (23 samples) and Pakistan (27 samples) identified lead contamination in 36.4%, 60.9% and 88.9% of samples respectively [32–34]. The other two studies, both from the USA in 1988 (ten samples) and 2005 (three samples), detected mercury and other trace elements in only a single sample in each study [30,31]. The Brazilian study also assessed API content and reported 31.1% (seven out of 23 samples) of calcium supplements were substandard with an API content below the specification range by Brazilian Pharmacopoeia [32].

### Antihypertensive medicines

We found 15 studies on the quality of antihypertensives (**Table 5**) [35–49]. Seven studies evaluated the quality of amlodipine- two studies from India and Nepal found no evidence of poor-quality amlodipine [36,38]. In contrast, a Nigerian study reported three of ten (30.0%) amlodipine samples were counterfeit as they were unregistered within regulatory bodies, and their API content did not comply with British Pharmacopoeia standard.[37] A study in Cambodia found seven amlodipine samples (8.9%) did not meet the API content and uniformity criteria according to the US Pharmacopoeia and British Pharmacopoeia [40]. Another two studies—one conducted in 10 different African countries [35] and the other in Nigeria [41]- reported 87 (28.5%) and 48 (24.6%) amlodipine samples as substandard, respectively, due to failure to meet API specification. An additional Nigerian study reported four (50.0%) samples as substandard: one sample (12.5%) did not meet the API specification and three (37.5%) samples failed in friability and hardness criteria [39].

Two studies assessed nifedipine quality- one from Germany reported 60.3% samples of nifedipine did not comply with the German Federal Health Authority and US Pharmacopoeia API specification, and 27.9% of packaged and 76.5% of unpackaged nifedipine did not pass the light stability test [43]. Whereas, the other study from Nigeria concluded that 78 nifedipine samples (76.5%) were of poor quality, with 31 (30.4%) samples failing to comply with API specifications of US Pharmacopoeia and International Pharmacopoeia, and 76 (74.5%) samples showing nitrophenylpyridine impurities above the acceptance range [42].

Three studies from Nigeria [45], Rwanda [44] and Brazil [46], assessed methyldopa quality. A Nigerian study and Brazilian study reported one (25.0%) sample and ten (50.0%) samples as substandard, due to not meeting the API specification [45,46]. The study from Brazil also reported that ten (50.0%) samples failed to meet the criteria of API content. In contrast, the Rwandan study reported all three samples cleared the physicochemical parameters tested immediately after their purchase [44].

Three studies assessed enalapril quality- two from Brazil [46,47] and one from Turkey [48]. One of the Brazilian studies (2011; 20 samples) reported 100% of samples were substandard due to failure to meet Brazilian Pharmacopoeia API specification [46]. The study from Turkey reported that all eight samples met the criteria of API content and friability, however, all samples failed in the hardness test [48]. The other Brazilian study (2014) detected contaminants (including aluminium, gold, bromine, cerium, cobalt, hafnium, lanthanum, antimony, scandium, samarium, thorium, zinc, chromium) in all five samples [47]. Only one study on the quality of hydralazine was identified, conducted in the USA in which 12 (75.0%) samples were found to be poor-quality due to presence of impurities resulting from degradation i.e. hydrazine, above the acceptance limit [49].

## Discussion

### Main findings

This is the first study to systematically evaluate the evidence on quality of medicines for HDP globally. We identified evidence of poor-quality of medicines for eight medicines—magnesium sulphate, aspirin, calcium supplements, and multiple antihypertensives, across high-, middle- and low-income countries. However, the majority of evidence come from studies in LMICs. The majority of studies (29/33) found evidence of poor-quality medicines used for the prevention and management of hypertensive disorders of pregnancy. The highest number of studies (15/33) assessed the quality of antihypertensives- almost all of which found evidence of poor-quality medicines, and the lowest number of studies assessed quality of calcium supplements and magnesium sulphate, five each.

### Strengths and limitations

To the best of our knowledge, this systematic review is the first to assess the quality of multiple medicines used for HDP globally. More than half the studies we assessed were methodologically of good quality, suggesting robustness in our findings. We aimed to minimise the possibility of bias by using a broad and systematic approach to identify eligible studies without any limitations on language, geographical region and year of publication. In addition, we searched for information in the grey literature to reduce publication bias and maximise data capture. Despite these efforts, there may be additional reports that are not publicly available or were otherwise inaccessible. For example, some national authority bodies may be reluctant to release study results showing a high prevalence of poor-quality medicines.

### Interpretation

In light of the examples of poor-quality HDP medicines that we identified—which crossed all medicine classes, diverse countries and multiple quality measures—we are concerned that this may be having a negative impact on maternal and newborn health. For example, a WHO review on medicine regulatory systems in 26 sub-Saharan African countries [51] reported that the presence of unregulated medicines in the market. Using such unauthorized medicines without quality assessment creates a profound risk for individual health by prolonging illness that leads to treatment failure, increasing morbidity and mortality. Failure to manage hypertension potentially increases the risk of developing preeclampsia/eclampsia potentially leading to premature delivery. This, in turn, impacts newborn development with long term effects from birth to adulthood including increased risk of developing hypertension, cardiac disease, metabolic disease and chronic renal disease [52]. The adverse consequences of poor-quality medicines for HDP specifically are difficult to predict. In low resource settings, there may be a number of barriers to high quality care, across the health systems. As such the adverse consequences of poor-quality medicines may initiate a flow on effect of adverse outcomes for women, as for example, they may lack access to appropriate care for HDPs, including access to routine antenatal care or diagnostic tools for preeclampsia, access to safe induction or caesarean delivery if required and high-quality neonatal cate if preterm delivery is needed to save the mother’s life. Thus, it is difficult to predict this flow on effect. Therefore, it is necessary to prevent, treat and manage HDP with high quality medicines. Insufficient API or degradation could result in inadequate dosing which could impair or reduce clinical benefit. For instance, poor-quality aspirin, calcium, antihypertensive medicines and magnesium sulphate [17–19,21–27,29–35,37,39–41,50] may mean that preeclampsia is more likely to develop, or worsen. It should be acknowledged, however, that the optimal (or minimum) dosing required for many obstetric medicines has not been established [53,54]. Contaminants—such as those reported for calcium, enalapril and magnesium sulphate [17,30–34,47]—are worrisome. In the absence of comprehensive toxicology evaluations, we can only speculate that these substances might jeopardise women’s and newborn’s health [55].

Poor-quality medicines can also reduce health workers’ confidence in the effectiveness of treatments, and otherwise impair public trust in the health system [11,14]. In addition, it affects public health due to poor health outcomes and also have adverse impacts due to additional health systems costs, wastage of resources, and increased out-of-pocket costs [11]. For instance, a study by Ozawa et al (2018) estimated the economic burden of substandard and falsified antimalarial and antibiotics in LMICs to range from $10 billion to $200 billion [10]. Currently, there are no cost-effectiveness studies on impact of poor-quality maternal medicines specifically, however, a study has shown the significant costs of caring for women with preeclampsia [56], which would be incurred if not effectively prevented or managed.

While this review was limited to medicines for HDP, quality issues affecting other medicines used in pregnant women have been identified. A 2020 systematic review on the quality of antenatal corticosteroids for preterm birth in LMIC [14] identified substandard dexamethasone samples due to inadequate API. A separate 2020 systematic review on the quality of multiple maternal medicines in LMIC identified 34 studies, with the highest failure rates reported for ergometrine, oxytocin and misoprostol [13]. A 2016 systematic review on the quality of oxytocin in LMICs identified poor quality of oxytocin samples due to insufficient API [15]. Combined with the evidence presented in the current study, there is clearly cause for concern that multiple medicines used in the management of leading causes of maternal and perinatal morbidity and mortality have quality issues.

Identifying precisely where and why quality issues arise within the manufacture and distribution pathways is key to developing strategies to improve medicine quality. However, most of the studies we identified did not assess important quality parameters, particularly physicochemical assays (dissolution, disintegration and friability assays). We also could not clearly identify the cause of low quality, as sample collection processes and locations were not described in most studies. It is thus difficult to draw conclusions whether poor-quality is attributable to manufacturing issues, storage conditions or a combination of both, based on currently available evidence. No studies reported information on how medicines had been stored in the sampling setting. Given that multiple studies reported on the presence of degradants or impurities, understanding storage conditions is particularly critical to addressing systematic causes of poor-quality medicines. Future research should comprehensively assess and report on all medicine quality parameters using reference methods, to facilitate a better understanding of the root causes of medicine quality issues. Studies should ideally follow WHO survey guidelines for post-marketing surveillance assessing medicines quality [57]. In addition, evaluating medicine quality from different outlets and points along the supply chain, and their current regulatory status, should also be performed. While it is acknowledged that resource capacity is limited, particularly in LMICs, we strongly encourage national regulatory authorities to implement robust pharmacovigilance and post-market surveillance programs to monitor and address poor-quality medicines, including for maternal health.

## Conclusion

This systematic review identified evidence of widespread quality issues in medicines for management of HDP. These medicines impose avoidable risks to the health of women and newborns and have adverse economic implications. In order to accelerate progress towards the Sustainable Development Goals, high-quality and effective medicines need to be easily accessible and affordable, in all countries. While further research is warranted, there is a clear need for rapid improvement in the regulation, procurement, storage and quality monitoring of these medicines.

## Supporting information

S1 Checklist PRISMA 2020 main checklist. (DOCX)

S1 Text Search strategy. (DOCX)

S2 Text Inclusion and exclusion criteria. (DOCX)
