# Supplementary material for: The quality of medicines for the prevention and management of hypertensive disorders of pregnancy: A systematic review
Source: PLOS Glob Public Health. 2024 Feb 27;4(2):e0002962. doi: 10.1371/journal.pgph.0002962 (PMC10898726; doi:10.1371/journal.pgph.0002962)
Supplement: S1 Text — (DOCX) [file pgph.0002962.s002.docx]

**S1_Search_strategy**

**Search strategy**

OVID MEDLINE Search

| # | Query | Results from 6 Oct 2022 |
| --- | --- | --- |
| 1 | hypertension/ or borderline hypertension/ or essential hypertension/ or hereditary hypertension/ or masked hypertension/ or maternal hypertension/ or systolic hypertension/ or white coat hypertension/ or HELLP syndrome/ or "eclampsia and preeclampsia"/ or eclampsia/ or preeclampsia/ | 287,827 |
| 2 | (hypertension or high blood pressure or HTN or hypertensive* or white coat syndrome or hemolysis elevated liver enzymes low platelet count syndrome or HELLP syndrome or pre-eclampsia or preclampsia or eclampsia).mp. | 556,087 |
| 3 | labetalol/ or (albetol or apo labetalol or apolabetalol or labetalol or labetalol hydrochloride or normodyne or presolol or trandate or amipress or ibidomide or labelol or labetolol).mp. | 2,515 |
| 4 | methyldopa/ or (aldomet or aldomine or alphamethyldopa or alpha methyl 3,4 dihydroxyphenylalanine or alpha methyl dopa or alpha methyldopa or alphadopa or amodopa or apo methyldopa or apomethyldopa or dopamet or dopegit or dopegyt or dopergit or emdopa or hydopa or meldopa or l alpha methyl dopa or l alpha methyldopa or l methyldopa or levo alpha methyldopa or levo methyldopa or medopren or methyl dopa or nudopa or presinol or presolisin or methyldopa or methyldopate or sembrina or alpha methyl l dopa or alpha methyldopa or alpha-methyl-l-dopa).mp. | 4,832 |
| 5 | nifedipine/ or (adalat or adefin or adipine or afeditab or aponifed or calcibloc or cordalat or cordipin or cordipine or corinfar or coronpin or depin or dipinkor or fenamon or nifangin or nifedipine or nifedipine monohydrochloride or procardia* or vasdalat or vascard).mp. | 23,315 |
| 6 | hydralazine/ or (apresoline or alphapress or apressin or aprezin or hydralazine or hydralazine hydrochloride or hydralazine mono hydrochloride or hydralazine monohydrochloride or hydrazinophthalazine or nepresol).mp. | 6,098 |
| 7 | amlodipine/ or (amlodipine or amlodipine maleate or amloc or amlodis or amlodipina or amlopin or amlor or istin or norvasc).mp. | 5,476 |
| 8 | enalapril maleate/ or enalapril/ or (enalapril or enalapril maleate or mk 421 or mk421 or renitec).mp. | 8,055 |
| 9 | acetylsalicylic acid/ or antithrombocytic agent/ or (acetylsalicylic acid or acetysal or acylpyrin or aspirin or antiplatelet agent* or anti-platelet agent* or antiplatelet drug* or anti-platelet drug*or acetosal or acetyl salicylate or acetyl salicylic acid or acetylsalicylate or antithrombocytic agent* or anti-thrombocytic agent* or colfarit or dispril or easprin or ecotrin or ecosprin or endosprin or magnecyl or micristin).mp. | 76,559 |
| 10 | calcium/ | 278,849 |
| 11 | (calcium adj (tablet* or supplement* or diet* supplement*)).mp. | 4,025 |
| 12 | magnesium sulfate/ or (heptahydrate magnesium sulfate or magnesium sulfate or magnesium sulphate).mp. | 7,194 |
| 13 | (antihypertens* or anti-hypertens* or antihypertensive drug* or anti hypertensive drug* or antihypertensive medicine* or anti hypertensive medicine* or antihypertensive* or cardiac drug* or cardiac medicine* or cardiac agent* or antihypertensive agent* or anti hypertensive agent* or cardioactive agent* or cardio active agent* or cardiovascular agent* or cardio vascular agent* or cardioactive drug* or cardio active drug* or cardio active medicine* or cardioactive medicine* or cardiovascular drug* or cardiovascular medicine* or cardio vascular drug* or cardio vascular medicine*).mp. | 120,400 |
| 14 | 1 or 2 | 556,087 |
| 15 | 3 or 4 or 5 or 6 or 7 or 8 or 9 or 10 or 11 or 12 or 13 | 510,152 |
| 16 | counterfeit drug/ or drug contamination/ | 12,296 |
| 17 | ((counterfeit* or fake or false or falsified or bogus or copied or fictitious or forged or fraudulent or phony or faked or imitation or pirate or pseudo or sham or adulterat* or contamin* or impur* or substandard or sub-standard or poor quality) adj6 (drug? or medicine* or product? or pharmaceutical* or agent? or pill? or tablet? or capsule? or dose? or bolus or lozenge? or pellet? or troche? or pilule? or medicament* or dosage* or medicinal* or substance* or supplement*)).mp. | 35,294 |
| 18 | drug quality/ or drug impurity/ or drug purity/ or drug quality defect/ | 11,768 |
| 19 | ((quality or purity) adj2 (drug? or medicine* or product? or pharmaceutical* or agent? or pill? or tablet? or capsule? or dose? or bolus or lozenge? or pellet? or troche? or pilule? or medicament* or medicinal* or dosage* or substance* or supplement*)).mp. | 11,934 |
| 20 | (defect* adj1 (drug* or medicine* or pharmaceutical* or pill? or tablet? or capsule? or dose? or bolus or lozenge? or pellet? or troche? or pilule? or medicament*)).mp. | 200 |
| 21 | 16 or 17 or 18 or 19 or 20 | 46,521 |
| 22 | 15 and 21 | 682 |

EMBASE Search

| **#** | **Query** | **Results from 6 Oct 2022** |
| --- | --- | --- |
| 1 | hypertension/ or borderline hypertension/ or essential hypertension/ or hereditary hypertension/ or masked hypertension/ or maternal hypertension/ or systolic hypertension/ or white coat hypertension/ or HELLP syndrome/ or "eclampsia and preeclampsia"/ or eclampsia/ or preeclampsia/ | 832,642 |
| 2 | (hypertension or high blood pressure or HTN or hypertensive* or white coat syndrome or hemolysis elevated liver enzymes low platelet count syndrome or HELLP syndrome or pre-eclampsia or preclampsia or eclampsia).mp. | 1,181,259 |
| 3 | 1 or 2 | 1,210,289 |
| 4 | labetalol/ or (albetol or apo labetalol or apolabetalol or labetalol or labetalol hydrochloride or normodyne or presolol or trandate or amipress or ibidomide or labelol or labetolol).mp. | 11,937 |
| 5 | methyldopa/ or (aldomet or aldomine or alphamethyldopa or alpha methyl 3,4 dihydroxyphenylalanine or alpha methyl dopa or alpha methyldopa or alphadopa or amodopa or apo methyldopa or apomethyldopa or dopamet or dopegit or dopegyt or dopergit or emdopa or hydopa or meldopa or l alpha methyl dopa or l alpha methyldopa or l methyldopa or levo alpha methyldopa or levo methyldopa or medopren or methyl dopa or nudopa or presinol or presolisin or methyldopa or methyldopate or sembrina or alpha methyl l dopa or alpha methyldopa or alpha-methyl-l-dopa).mp. | 18,545 |
| 6 | nifedipine/ or (adalat or adefin or adipine or afeditab or aponifed or calcibloc or cordalat or cordipin or cordipine or corinfar or coronpin or depin or dipinkor or fenamon or nifangin or nifedipine or nifedipine monohydrochloride or procardia* or vasdalat or vascard).mp. | 53,823 |
| 7 | hydralazine/ or (apresoline or alphapress or apressin or aprezin or hydralazine or hydralazine hydrochloride or hydralazine mono hydrochloride or hydralazine monohydrochloride or hydrazinophthalazine or nepresol).mp. | 22,275 |
| 8 | amlodipine/ or (amlodipine or amlodipine maleate or amloc or amlodis or amlodipina or amlopin or amlor or istin or norvasc).mp. | 29,508 |
| 9 | enalapril maleate/ or enalapril/ or (enalapril or enalapril maleate or mk 421 or mk421 or renitec).mp. | 32,348 |
| 10 | acetylsalicylic acid/ or antithrombocytic agent/ or (acetylsalicylic acid or acetysal or acylpyrin or aspirin or antiplatelet agent* or anti-platelet agent* or antiplatelet drug* or anti-platelet drug*or acetosal or acetyl salicylate or acetyl salicylic acid or acetylsalicylate or antithrombocytic agent* or anti-thrombocytic agent* or colfarit or dispril or easprin or ecotrin or ecosprin or endosprin or magnecyl or micristin).mp. | 290,626 |
| 11 | calcium/ | 346,446 |
| 12 | (calcium adj (tablet* or supplement* or diet* supplement*)).mp. | 6,870 |
| 13 | magnesium sulfate/ or (heptahydrate magnesium sulfate or magnesium sulfate or magnesium sulphate).mp. | 21,038 |
| 14 | (antihypertens* or anti-hypertens*).mp. | 169,935 |
| 15 | 4 or 5 or 6 or 7 or 8 or 9 or 10 or 11 or 12 or 13 or 14 | 906,152 |
| 16 | counterfeit drug/ or drug contamination/ | 10,232 |
| 17 | ((counterfeit* or fake or false or falsified or bogus or copied or fictitious or forged or fraudulent or phony or faked or imitation or pirate or pseudo or sham or adulterat* or contamin* or impur* or substandard or sub-standard or poor quality) adj6 (drug? or medicine* or product? or pharmaceutical* or agent? or pill? or tablet? or capsule? or dose? or bolus or lozenge? or pellet? or troche? or pilule? or medicament* or dosage* or medicinal* or substance* or supplement*)).mp. | 54,529 |
| 18 | drug quality/ or drug impurity/ or drug purity/ or drug quality defect/ | 23,764 |
| 19 | ((quality or purity) adj2 (drug? or medicine* or product? or pharmaceutical* or agent? or pill? or tablet? or capsule? or dose? or bolus or lozenge? or pellet? or troche? or pilule? or medicament* or medicinal* or dosage* or substance* or supplement*)).mp. | 39,428 |
| 20 | (defect* adj1 (drug* or medicine* or pharmaceutical* or pill? or tablet? or capsule? or dose? or bolus or lozenge? or pellet? or troche? or pilule? or medicament*)).mp. | 293 |
| 21 | 16 or 17 or 18 or 19 or 20 | 90,864 |
| 22 | 3 and 15 and 21 | 877 |

Cinahl search

| **#** | **Query** | **Results from 6 Oct 2022** |
| --- | --- | --- |
| 1 | (MH "Hypertension/DT") OR (MH "Essential Hypertension/DT") OR (MH "Hypertension, Isolated Systolic") OR (MH "Hypertension, White Coat") OR (MH "Masked Hypertension") OR (MH "Pregnancy-Induced Hypertension") OR (MH "Eclampsia") OR (MH "Pre-Eclampsia") | 28,329 |
| 2 | hypertension/ or borderline hypertension/ or essential hypertension/ or hereditary hypertension/ or masked hypertension/ or maternal hypertension/ or systolic hypertension/ or white coat hypertension/ or HELLP syndrome/ or "eclampsia and preeclampsia"/ or eclampsia/ or preeclampsia/ | 144,802 |
| 3 | #1 OR #2 | 144,805 |
| 4 | (MH "Labetalol") | 333 |
| 5 | labetalol/ or (albetol or apo labetalol or apolabetalol or labetalol or labetalol hydrochloride or normodyne or presolol or trandate or amipress or ibidomide or labelol or labetolol/ | 492 |
| 6 | #4 OR #5 | 492 |
| 7 | (MH "Methyldopa") | 176 |
| 8 | methyldopa/ or (aldomet or aldomine or alphamethyldopa or alpha methyl 3,4 dihydroxyphenylalanine or alpha methyl dopa or alpha methyldopa or alphadopa or amodopa or apo methyldopa or apomethyldopa or dopamet or dopegit or dopegyt or dopergit or emdopa or hydopa or meldopa or l alpha methyl dopa or l alpha methyldopa or l methyldopa or levo alpha methyldopa or levo methyldopa or medopren or methyl dopa or nudopa or presinol or presolisin or methyldopa or methyldopate or sembrina or alpha methyl l dopa or alpha methyldopa or alpha-methyl-l-dopa/ | 300 |
| 9 | #7 OR #8 | 300 |
| 10 | (MH "Nifedipine") | 1,072 |
| 11 | nifedipine/ or (adalat or adefin or adipine or afeditab or aponifed or calcibloc or cordalat or cordipin or cordipine or corinfar or coronpin or depin or dipinkor or fenamon or nifangin or nifedipine or nifedipine monohydrochloride or procardia* or vasdalat or vascard/ | 1,695 |
| 12 | #10 OR #11 | 1,695 |
| 13 | (MH "Hydralazine") | 414 |
| 14 | hydralazine/ or (apresoline or alphapress or apressin or aprezin or hydralazine or hydralazine hydrochloride or hydralazine mono hydrochloride or hydralazine monohydrochloride or hydrazinophthalazine or nepresol/ | 703 |
| 15 | #13 OR #14 | 703 |
| 16 | (MH "Amlodipine") | 987 |
| 17 | amlodipine/ or (amlodipine or amlodipine maleate or amloc or amlodis or amlodipina or amlopin or amlor or istin or Norvasc/ | 1,703 |
| 18 | #16 OR #17 | 1,703 |
| 19 | (MH "Enalapril") | 795 |
| 20 | enalapril maleate/ or enalapril/ or (enalapril or enalapril maleate or mk 421 or mk421 or renitec/ | 1,183 |
| 21 | #19 OR 20 | 1,183 |
| 22 | (MH "Aspirin") | 12,789 |
| 23 | acetylsalicylic acid/ or antithrombocytic agent/ or (acetylsalicylic acid or acetysal or acylpyrin or aspirin or antiplatelet agent* or anti-platelet agent* or antiplatelet drug* or anti-platelet drug*or acetosal or acetyl salicylate or acetyl salicylic acid or acetylsalicylate or antithrombocytic agent* or anti-thrombocytic agent* or colfarit or dispril or easprin or ecotrin or ecosprin or endosprin or magnecyl or micristin/ | 20,538 |
| 24 | #22 OR #23 | 20,538 |
| 25 | (MH "Dietary Supplements") OR (MH "Dietary Supplementation") OR (MH "Calcium, Dietary") | 50,238 |
| 26 | (calcium adj (tablet* or supplement* or diet* supplement*)/ | 37 |
| 27 | #25 OR #26 | 50,243 |
| 28 | (MH "Magnesium Sulfate") | 1,702 |
| 29 | magnesium sulfate/ or (heptahydrate magnesium sulfate or magnesium sulfate or magnesium sulphate)/ | 2,294 |
| 30 | #28 OR #29 | 2,294 |
| 31 | #3 OR #6 OR #9 OR #12 OR #15 OR #18 OR #21 OR #24 OR #27 OR #30 | 78,201 |
| 32 | (MH "Drug Quality") | 4,385 |
| 33 | counterfeit drug/ or drug contamination/ | 2,351 |
| 34 | (counterfeit drug* or counterfeit medicine* or fake drug* or falsified drug* or falsified medicine* or fake medicine* or drug* adulteration* or chemical drug contamination* or drug* contamination* or microbial drug contamination* or physical drug contamination* or adulteration adj1 (drug*) or drug impurit*)/ | 2,524 |
| 35 | ((quality or purity) adj2 (drug? or medicine* or product? or pharmaceutical* or agent? or pill? or tablet? or capsule? or dose? or bolus or lozenge? or pellet? or troche? or pilule? or medicament* or medicinal* or dosage* or substance* or supplement*))/ | 8 |
| 36 | #32 OR #33 OR #34 OR #35 | 2,587 |
| 37 | #3 OR #31 | 216,387 |
| 38 | #37 AND #36 | 198 |

ProQuest search

| **#** | **Query** | **Results from 6 Oct 2022** |
| --- | --- | --- |
| 1 | (labetalol OR albetol OR “apo labetalol” OR apolabetalol OR labetalol OR “labetalol hydrochloride” OR normodyne OR presolol OR trandate OR amipress OR ibidomide OR labelol OR labetolol OR methyldopa OR aldomet OR aldomine OR alphamethyldopa OR “alpha methyl 3,4 dihydroxyphenylalanine” OR “alpha methyl dopa” OR “alpha methyldopa” OR alphadopa OR amodopa OR “apo methyldopa” OR apomethyldopa OR dopamet OR dopegit OR dopegyt OR dopergit OR emdopa OR hydopa OR meldopa OR “l alpha methyl dopa” OR “l alpha methyldopa” OR “l methyldopa” OR “levo alpha methyldopa” OR “levo methyldopa” OR medopren OR “methyl dopa” OR nudopa OR presinol OR presolisin OR methyldopa OR methyldopate OR sembrina OR “alpha methyl l dopa” OR “alpha methyldopa” OR “alpha-methyl-l-dopa” OR nifedipine OR adalat OR adefin OR adipine OR afeditab OR aponifed OR calcibloc OR cordalat OR cordipin OR cordipine OR corinfar OR coronpin OR depin OR dipinkor OR fenamon OR nifangin OR nifedipine OR “nifedipine monohydrochloride” OR procardia* OR vasdalat OR vascard OR hydralazine OR apresoline OR alphapress OR apressin OR aprezin OR hydralazine OR “hydralazine hydrochloride” OR “hydralazine mono hydrochloride” OR “hydralazine monohydrochloride” OR hydrazinophthalazine OR nepresol OR amlodipine OR amlodipine OR “amlodipine maleate” OR amloc OR amlodis OR amlodipina OR amlopin OR amlor OR istin OR norvasc OR “enalapril maleate” OR enalapril OR enalapril OR “mk 421” OR mk421 OR renitec OR “acetylsalicylic acid” OR “antithrombocytic agent” OR acetysal OR acylpyrin OR aspirin OR “antiplatelet agent*” OR “anti-platelet agent*” OR “antiplatelet drug*” OR “anti-platelet drug*” or acetosal OR “acetyl salicylate” OR “acetyl salicylic acid” OR acetylsalicylate OR “antithrombocytic agent*” OR “anti-thrombocytic agent*” OR colfarit OR dispril OR easprin OR ecotrin OR ecosprin OR endosprin OR magnecyl OR micristin OR calcium OR “calcium tablet*” OR “calcium supplement*” OR “calcium diet* supplement*” OR “magnesium sulfate” OR “heptahydrate magnesium sulfate” OR “magnesium sulphate” OR “antihypertens*” OR “anti-hypertens*” OR “antihypertensive drug*” OR “anti hypertensive drug*” OR “antihypertensive medicine*” OR “anti hypertensive medicine*” OR antihypertensive* OR “cardiac drug*” OR “cardiac medicine*” OR “cardiac agent*” OR “antihypertensive agent*” OR “anti hypertensive agent*” OR “cardioactive agent*” OR “cardio active agent*” OR “cardiovascular agent*” OR “cardio vascular agent*” OR “cardioactive drug*” OR “cardio active drug*” OR “cardio active medicine*” OR “cardioactive medicine*” OR “cardiovascular drug*” OR “cardiovascular medicine*” OR “cardio vascular drug*” OR “cardio vascular medicine*”) AND (“Counterfeit Drug*” OR “Drug Contamination” OR “drug quality” OR “quality control” OR “drug monitoring” OR “substandard drugs” OR “drug* storage” OR “drug* stability” OR “active pharmaceutical ingredient*” OR “active pharmacological ingredient*” OR “drug* potenc*” OR “drug* sterility” OR “medication error*” OR “quality control” OR “substandard drug*” OR “unregistered drug*” OR “unregistered medicine*” OR “substandard medicine*” OR “drug* degradation” OR “medicine* degradation” OR “medicine* sterility” OR “drug* purity” OR “medicine* purity” OR “drug* physicochemical change*” OR “drug* physicochemical phenomena” OR “drug* solubility” OR “medicine* solubility” OR “drug* dissolution” OR “medicine* dissolution” OR “counterfeit medicine*” OR “fake drug*” OR “falsified drug*” OR “falsified medicine*” OR “fake medicine*” OR “drug* adulteration*” OR “chemical drug contamination*” OR “drug* contamination*” OR “microbial drug contamination*” OR “physical drug contamination*” OR “adulterated drugs” OR “drug impurit*”) | 5,270 |

Cochrane Library search

| **#** | **Query** | **Results from 6 Oct 2022** |
| --- | --- | --- |
| 1 | MeSH: Hypertension | 24,941 |
| 2 | (hypertension NEXT (high blood pressure*)):ti,ab,kw OR (hypertension NEXT (gestational*)):ti,ab,kw OR (hypertension NEXT (pregnancy induce*)):ti,ab,kw OR (hypertension NEXT (pregnancy transient)):ti,ab,kw OR (hypertension NEXT (preeclampisa or eclampsia or hellp syndrome)):ti,ab,kw | 2,880 |
| 3 | #1 OR #2 | 25,197 |
| 4 | MeSH: Labetalol | 443 |
| 5 | (labetalol NEXT (Apo* labetalol or normodyne or albetol)):ti,ab,kw OR (Labetalol NEXT (presolol or R,R labetalol* or dilevalol)):ti,ab,kw | 89 |
| 6 | #4 OR #5 | 486 |
| 7 | MeSH: Methyldopa | 353 |
| 8 | (Methyldopa NEXT (dopamet or methyldopate or hydopa or apo*methyldopa or aldomet or sembrina or alpha*methyldopa or dopegyt or dopergit or meldopa)):ti,ab,kw | 5 |
| 9 | #7 OR #8 | 352 |
| 10 | MeSH: Nifedipine | 2,239 |
| 11 | (Nifedipine NEXT (adalat or adefin or adipine or afeditab or aponified or calcibloc or cordalat or cordipin* or coinfar or coronpin or depin or dipinkor or fenamon or nifangin or nifedipine monohydrochloride or nepresol or hydrazinophthalazine)):ti,ab,kw | 71 |
| 12 | #10 OR #11 | 2,263 |
| 13 | MeSH: Amlodipine | 1,786 |
| 14 | (Amlodipine NEXT (amlodipine maleate or amloc or amlodis or amlodipina or amlopin or amlor or istin or norvasc)):ti,ab,kw | 72 |
| 15 | #13 OR #14 | 1,817 |
| 16 | MeSH: Hydralazine | 340 |
| 17 | (Hydralazine NEXT (apresoline or alphapress or apressin or aprezin or hydralazine hydrochloride or hydralazine mono*hydrochloride or nepresol)):ti,ab,kw | 12 |
| 18 | #16 OR #17 | 347 |
| 19 | MeSH: Enalapril | 1,822 |
| 20 | (Enalapril NEXT (enalapril maleate or mk*421 or renitec)):ti,ab,kw | 203 |
| 21 | #19 OR #20 | 1,949 |
| 22 | MeSH: Aspirin | 7,051 |
| 23 | (Aspirin NEXT (acetyl*salicylic acid or acetylsal or acylpyrin)):ti,ab,kw OR (acetylsalicylate NEXT (anti*platelet agent* or anti*platelet drug* or acetosal or acetyl*salicylate or anti*thrombocytic agent* or colfarit or dispril or easprin or ecotrin or ecosprin or endosprin or magnecyl or micristin)):ti,ab,kw | 80 |
| 24 | #22 OR #23 | 6,981 |
| 25 | MeSH: Magnesium sulfate | 1,201 |
| 26 | (Magnesium sulphate NEXT (heptahydrate magnesium sulphate or heptahydrate magnesium sulfate)):ti,ab,kw | 77 |
| 27 | #25 OR #26 | 1,227 |
| 28 | MeSH: Calcium | 4,065 |
| 29 | (Calcium NEXT (calcium supplement* or calcium diet*)):ti,ab,kw | 3,088 |
| 30 | #28 OR #29 | 6,297 |
| 31 | MeSH: Drug contamination | 71 |
| 32 | (Drug contamination NEXT (counterfeit drug* or drug* quality or drug impurity or drug purity or drug quality defect or poor*quality or active pharmaceutical ingredient* or stability or sterility)):ti,ab,kw | 18 |
| 33 | #31 OR #32 | 83 |
| 34 | #6 OR #9 OR #12 OR #15 OR #18 OR #21 OR #24 OR #27 OR #30 | 21,062 |
| 35 | #3 and 34 | 3,826 |
| 36 | #33 or #34 | 21,144 |
| 37 | #35 and #36 | 3,716 |
