# Supplementary material for: The quality of medicines for the prevention and management of hypertensive disorders of pregnancy: A systematic review
Source: PLOS Glob Public Health. 2024 Feb 27;4(2):e0002962. doi: 10.1371/journal.pgph.0002962 (PMC10898726; doi:10.1371/journal.pgph.0002962)
Supplement: S2 Text — (DOCX) [file pgph.0002962.s003.docx]

**S1_Inclusion and exclusion criteria**

**List of inclusion and exclusion criteria**

**Inclusion criteria**

1. We considered any studies assessing the quality of medicines used for the prevention and management of hypertensive disorders of pregnancy, using any valid laboratory methods, to be eligible for this systematic review.
2. We specified nine medicines- calcium, aspirin, labetalol, nifedipine, methyldopa, hydralazine, amlodipine, enalapril and magnesium sulphate for this review, on the basis of current World Health Organisation (WHO) and National Institute for Clinical and Healthcare Excellence (NICE) guidelines for hypertensive disorders of pregnancy.
3. Any peer-reviewed studies, reports and grey literature describing the quality of medicines for hypertensive disorders of pregnancy.
4. Studies were eligible regardless of their design (observational or interventional) or level of sample collection sites (hospitals, clinics, pharmacies, local medical stores, informal vendors or markets, or central distributors like warehouse, major distributors or central medical stores).
5. No limits were placed on samples size, publication date, geographical location or language.

**Exclusion criteria**

1. Studies assessing the quality of medicines creating conditions to test the effects of heat, light or other factors were excluded.
2. Studies conducting in animal and human were excluded for this review.
3. Any bioequivalence studies were also excluded.
